# REDISCOVER guidelines for borderline-resectable and locally advanced pancreatic cancer: management algorithm, unanswered questions, and future perspectives

**DOI:** 10.1007/s13304-024-01860-0

**Published:** 2024-04-29

**Authors:** Ugo Boggi, Emanuele F. Kauffmann, Niccolò Napoli, S. George Barreto, Marc G. Besselink, Giuseppe K. Fusai, Thilo Hackert, Mohammad Abu Hilal, Giovanni Marchegiani, Roberto Salvia, Shailesh V. Shrikhande, Mark Truty, Jens Werner, Christopher Wolfgang, Elisa Bannone, Giovanni Capretti, Alice Cattelani, Alessandro Coppola, Alessandro Cucchetti, Davide De Sio, Armando Di Dato, Giovanna Di Meo, Claudio Fiorillo, Cesare Gianfaldoni, Michael Ginesini, Camila Hidalgo Salinas, Quirino Lai, Mario Miccoli, Roberto Montorsi, Michele Pagnanelli, Andrea Poli, Claudio Ricci, Francesco Sucameli, Domenico Tamburrino, Virginia Viti, John Cameron, Pierre-Alain Clavien, Horacio J. Asbun, the REDISCOVER guidelines group, Pietro F. Addeo, Sergio Alfieri, Philippe Bachellier, Gianluca Baiocchi, Gianpaolo Balzano, Linda Barbarello, Alberto Brolese, Juli Busquets, Giovanni Butturini, Fabio Caniglia, Damiano Caputo, Riccardo Casadei, Xi Chunhua, Ettore Colangelo, Andrea Coratti, Francesca Costa, Francesco Crafa, Raffaele Dalla Valle, Luciano De Carlis, Roeland F de Wilde, Marco Del Chiaro, Fabrizio Di Benedetto, Pierluigi Di Sebastiano, Safi Dokmak, Melissa Hogg, Vyacheslav I. Egorov, Giorgio Ercolani, Giuseppe Maria Ettorre, Massimo Falconi, Giovanni Ferrari, Alessandro Ferrero, Marco Filauro, Alessandro Giardino, Gian Luca Grazi, Salvatore Gruttadauria, Jakob R. Izbicki, Elio Jovine, Matthew Katz, Tobias Keck, Igor Khatkov, Gozo Kiguchi, David Kooby, Hauke Lang, Carlo Lombardo, Giuseppe Malleo, Marco Massani, Vincenzo Mazzaferro, Riccardo Memeo, Yi Miao, Kohei Mishima, Carlo Molino, Yuichi Nagakawa, Masafumi Nakamura, Bruno Nardo, Fabrizio Panaro, Claudio Pasquali, Vittorio Perrone, Elena Rangelova, Long Riu, Renato Romagnoli, Raffaele Romito, Edoardo Rosso, Richard Schulick, Ajith K. Siriwardena, Marcello Spampinato, Oliver Strobel, Mario Testini, Roberto Troisi, Faik G. Uzunoglo, Roberto Valente, Luigi Veneroni, Alessandro Zerbi, Emilio Vicente, Fabio Vistoli, Marco Vivarelli, Go Wakabayashi, Giacomo Zanus, Amer Zureikat, Nicholas J. Zyromski, Roberto Coppola, Vito D’Andrea, José Davide, Christos Dervenis, Isabella Frigerio, Kevin C. Konlon, Fabrizio Michelassi, Marco Montorsi, William Nealon, Nazario Portolani, Donzília Sousa Silva, Giuseppe Bozzi, Viviana Ferrari, Maria G. Trivella, Piero Boraschi, Daniela Campani, Carla Cappelli, Roberto Cioni, Massimo Dominici, Irene Esposito, Maria A. Gambacorta, Emanuele Marciano, Gianluca Masi, Alessio Morganti, Massimiliano Mutignani, Emanuele Neri, Fabiola Paiar, Michele Reni, Maria Isabella Rotondo, Nicola Silvestris, Giampaolo Tortora, Enrico Vasile, Duccio Volterran

**Affiliations:** 1 Division of General and Transplant Surgery, University of Pisa, Via Savi 10, 56126 Pisa, PI Italy; 2 College of Medicine and Public Health, Flinders University, Adelaide, South Australia Australia; 3 Division of Surgery and Perioperative Medicine, Flinders Medical Center, Beadfor Park, Australia; 4 Department of Surgery, Amsterdam UMC, Location University of Amsterdam, Amsterdam, The Netherlands; 5 Cancer Center Amsterdam, Amsterdam, The Netherlands; 6 HPB and Liver Transplant Unit, Royal Free Hospital, London, UK; 7 Department of General, Visceral and Thoracic Surgery, University Hospital Hamburg-Eppendorf, Hamburg, Germany; 8 Department of Surgery, Poliambulanza Foundation Hospital, Brescia, Italy; 9 Hepatopancreatobiliary and Liver Transplant Surgery, Department of Surgery, Oncology and Gastroenterology, DiSCOG, University of Padua, Padua, Italy; 10 General and Pancreatic Surgery Unit, Pancreas Institute, University of Verona, Verona, Italy; 11 Tata Memorial Centre, Gastrointestinal and HPB Service, Tata Memorial Hospital, Homi Bhabha National Institute, Mumbai, Maharashtra, India; 12 Department of Surgery, Division of Hepatobiliary and Pancreas Surgery, Mayo Clinic Rochester, Rochester, MN USA; 13 Department of General, Visceral, and Transplant Surgery, LMU, University of Munich, Munich, Germany; 14 Department of Surgery, The NYU Grossman School of Medicine and NYU Langone Health, New York, NY USA; 15 IRCCS Humanitas Research Hospital, Rozzano, Milan Italy; 16 Department of Surgery, Sapienza University of Rome, Rome, Italy; 17 Department of Medical and Surgical Sciences - DIMEC, Alma Mater Studiorum Università di Bologna, Bologna, Italy; 18 Gemelli Pancreatic Center, CRMPG (Advanced Pancreatic Research Center), Fondazione Policlinico Universitario “Agostino Gemelli” IRCCS, Università Cattolica del Sacro Cuore, Rome, Italy; 19 Department of Precision and Regenerative Medicine and Ionian Area (DiMePre-J), University of Bari, Bari, Italy; 20 Kellogg College, University of Oxford, Oxford, UK; 21 Department of General and Specialty Surgery, Sapienza University of Rome, AOU Policlinico Umberto I of Rome, Rome, Italy; 22 Department of Clinical and Experimental Medicine, University of Pisa, Pisa, Italy; 23 Division of Pancreatic Surgery, Department of Internal Medicine and Surgery (DIMEC), Alma Mater Studiorum, University of Bologna, IRCCS, Azienda Ospedaliero-Universitaria di Bologna (IRCCS AOUBO), Bologna, Italy; 24 Division of Pancreatic Surgery, Pancreas Translational and Clinical Research Center, IRCCS San Raffaele Scientific Institute, Vita-Salute University, Milan, Italy; 25 Department of Surgery, John Hopkins University School of Medicine, Baltimore, MD USA; 26 Department of Surgery and Transplantation, University Hospital Zurich, University of Zurich, Zurich, Switzerland; 27 Division of Hepatobiliary and Pancreas Surgery, Miami Cancer Institute, Miami, FL USA

**Keywords:** Pancreatic ductal adenocarcinoma, Pancreatic cancer, Locally advanced pancreatic cancer, Borderline resectable pancreatic cancer, REDISCOVER guidelines, REDISCOVER registry

## Abstract

The REDISCOVER guidelines present 34 recommendations for the selection and perioperative care of borderline-resectable (BR-PDAC) and locally advanced ductal adenocarcinoma of the pancreas (LA-PDAC). These guidelines represent a significant shift from previous approaches, prioritizing tumor biology over anatomical features as the primary indication for resection. Condensed herein, they provide a practical management algorithm for clinical practice. However, the guidelines also highlight the need to redefine LA-PDAC to align with modern treatment strategies and to solve some contradictions within the current definition, such as grouping "difficult" and "impossible" to resect tumors together. Furthermore, the REDISCOVER guidelines highlight several areas requiring urgent research. These include the resection of the superior mesenteric artery, the management strategies for patients with LA-PDAC who are fit for surgery but unable to receive multi-agent neoadjuvant chemotherapy, the approach to patients with LA-PDAC who are fit for surgery but demonstrate high serum Ca 19.9 levels even after neoadjuvant treatment, and the optimal timing and number of chemotherapy cycles prior to surgery. Additionally, the role of primary chemoradiotherapy versus chemotherapy alone in LA-PDAC, the timing of surgical resection post-neoadjuvant/primary chemoradiotherapy, the efficacy of ablation therapies, and the management of oligometastasis in patients with LA-PDAC warrant investigation. Given the limited evidence for many issues, refining existing management strategies is imperative. The establishment of the REDISCOVER registry (https://rediscover.unipi.it/) offers promise of a unified research platform to advance understanding and improve the management of BR-PDAC and LA-PDAC.

## Introduction

Pancreatic ductal adenocarcinoma (PDAC) is recognized for its aggressive biological behavior, often resulting in a fatal outcome for the majority of affected individuals. This heightened mortality rate is predominantly attributed to early hematogenous spread and intrinsic resistance to conventional oncological treatments. Consequently, distant metastases are frequently detectable at the time of diagnosis in approximately 60% of patients, rendering curative resection of the primary tumor unattainable. Tumor size stands out as a key prognostic factor in resected PDAC. While larger tumor sizes increase the risk of distant metastases, such metastases can also manifest in up to one-third of patients with tumors measuring 0.5 cm or less. Given the significant morbidity and mortality associated with pancreatic resection, surgical intervention is generally reserved for patients with localized disease [1–8].

Localized PDAC can be classified into three subgroups: resectable, borderline resectable (BR-PDAC), and locally advanced (LA-PDAC). These categories are based on the degree of vascular involvement and the probability of achieving a margin-negative resection. BR-PDAC and LA-PDAC comprise approximately one-third of all PDAC cases. Recently, the concept of anatomical resectability has been expanded to include biologic resectability, primarily assessed by levels of Ca 19-9, and conditional resectability, which considers the patient's overall health status and any comorbidities. The contemporary approach to resectability in pancreatic cancer integrates these concepts into what is commonly referred to as the A, B, C criteria [9].

Recent advancements in chemotherapy have shifted the focus towards the biologic aspect of resectability, leading to the emergence of "prognosis-based resectability", also known as conversion surgery [10, 11]. A study employing an intention-to-treat analysis of neoadjuvant FOLFIRINOX for PDAC demonstrated that surgical exploration and the attainment of negative margins at pathology were equally achievable across all anatomical resectability categories [12]. Similarly, the NORPATC-2 trial corroborated that the response to preoperative chemotherapy remains unaffected by local tumor growth. Notably, the necessity for vascular resection was consistent across the three resectability categories, and both BR-PDAC and LA-PDAC exhibited comparable survival rates. Moreover, surgical resection was found to enhance survival outcomes relative to continued medical treatment [13].

The recent REDISCOVER guidelines have issued a consensus document endorsing a prognosis-based approach to resection in PDAC over an anatomy-based approach, while also offering insights into perioperative care specifics. However, the level of evidence supporting these recommendations was predominantly low, and several issues could not be endorsed due to insufficient evidence or reservations about incorporating avant-garde strategies into the guideline document [14].

This second report from the REDISCOVER consensus meeting aims to introduce a management algorithm for BR-PDAC and LA-PDAC Additionally, it addresses the questions that were not approved, highlighting the most crucial areas for future research.

The REDISCOVER guidelines were an initiative of the Italian Society of Surgery, endorsed by the Pancreas Club Inc. and the European-African Hepato-Pancreato-Biliary Association (blue seal).

## Methods

**Fig. 1:**
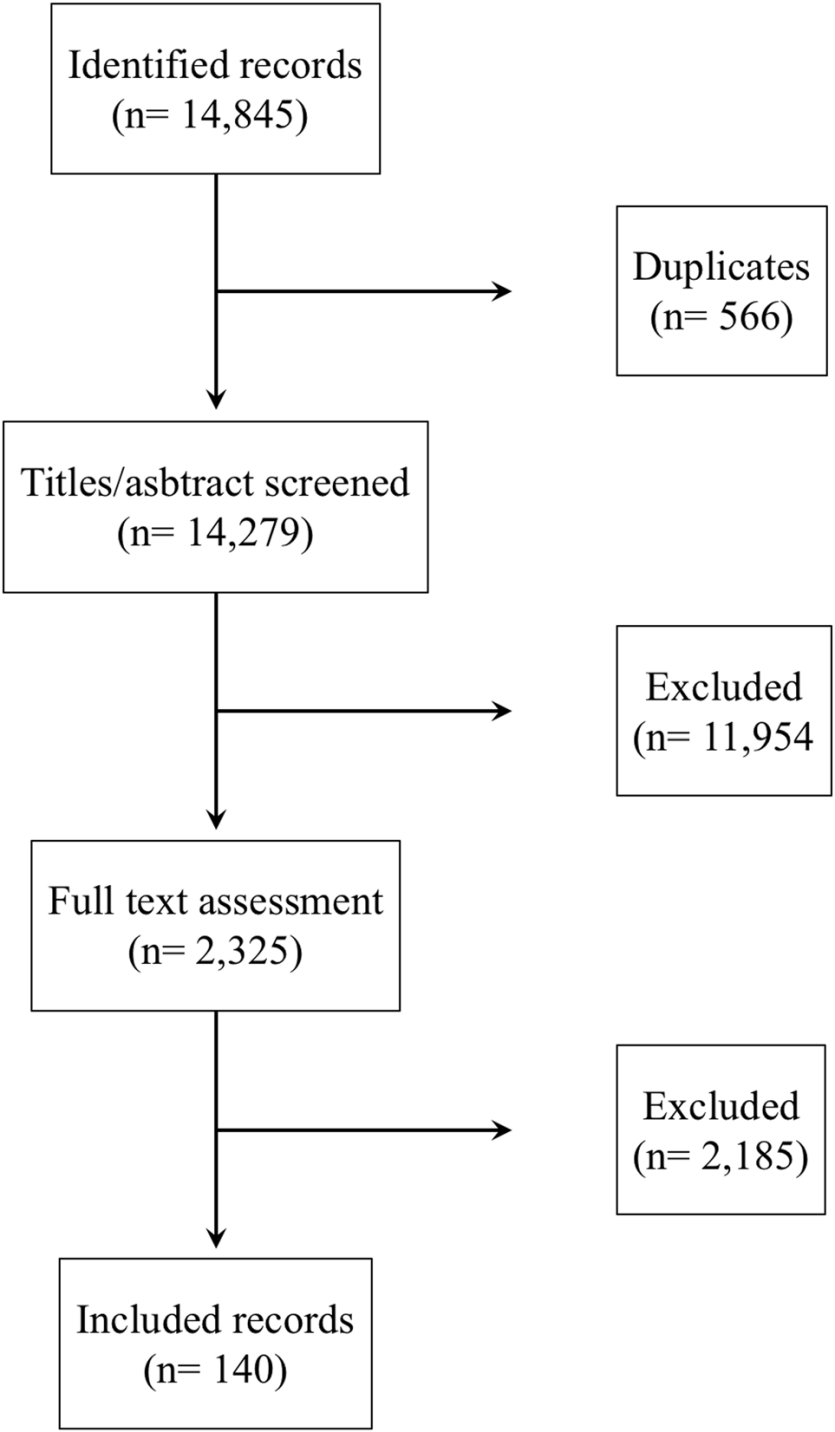
Flowchart of systematic literature review (reproduced from Ann Surg. 2024 Feb 26. 10.1097/SLA.0000000000006248)

**Fig. 2:**
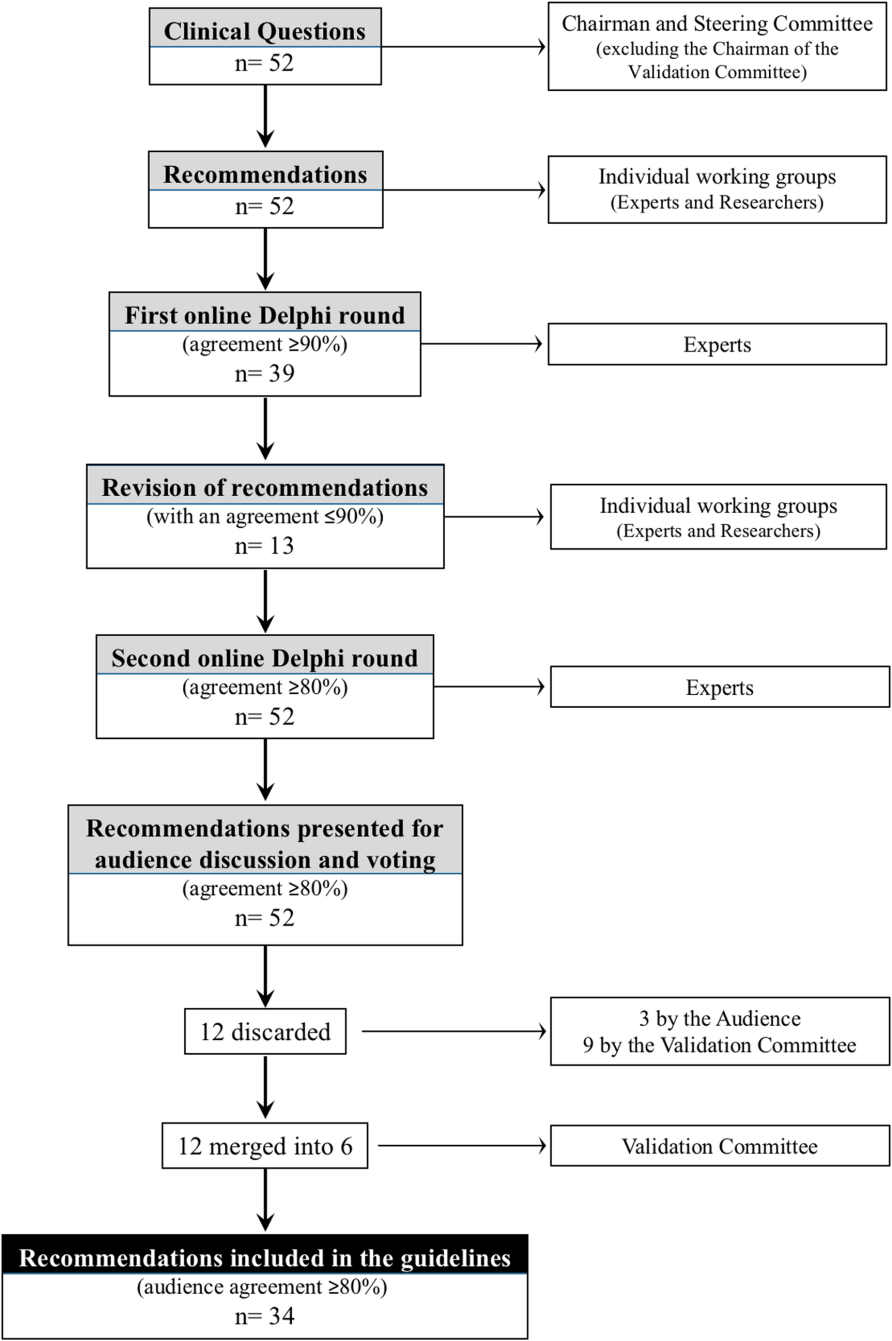
Flowchart of the guideline process (reproduced from Ann Surg. 2024 Feb 26. 10.1097/SLA.0000000000006248)

The REDISCOVER guidelines encompassed 34 recommendations that received approval during the final consensus conference held in Pisa, Italy, on September 17 and 18, 2023. The comprehensive PRISMA flowchart detailing the literature review and the consensus conference workflow is depicted in Figs. 1 and 2, respectively.

Utilizing these endorsed recommendations, we formulated a management algorithm tailored for the perioperative care of patients diagnosed with BR- and LA-PDAC. The clinical questions that did not garner approval were scrutinized to identify the most pressing areas requiring further clinical investigation.

## Results

The consensus conference witnessed participation from 136 attendees spanning 18 countries, including Australia, Austria, China, Italy, England, France, Germany, Greece, India, Ireland, Japan, Portugal, Russia, Spain, Sweden, Switzerland, The Netherlands, and the USA. The total audience count surpassed 150 participants.

**Fig. 3:**
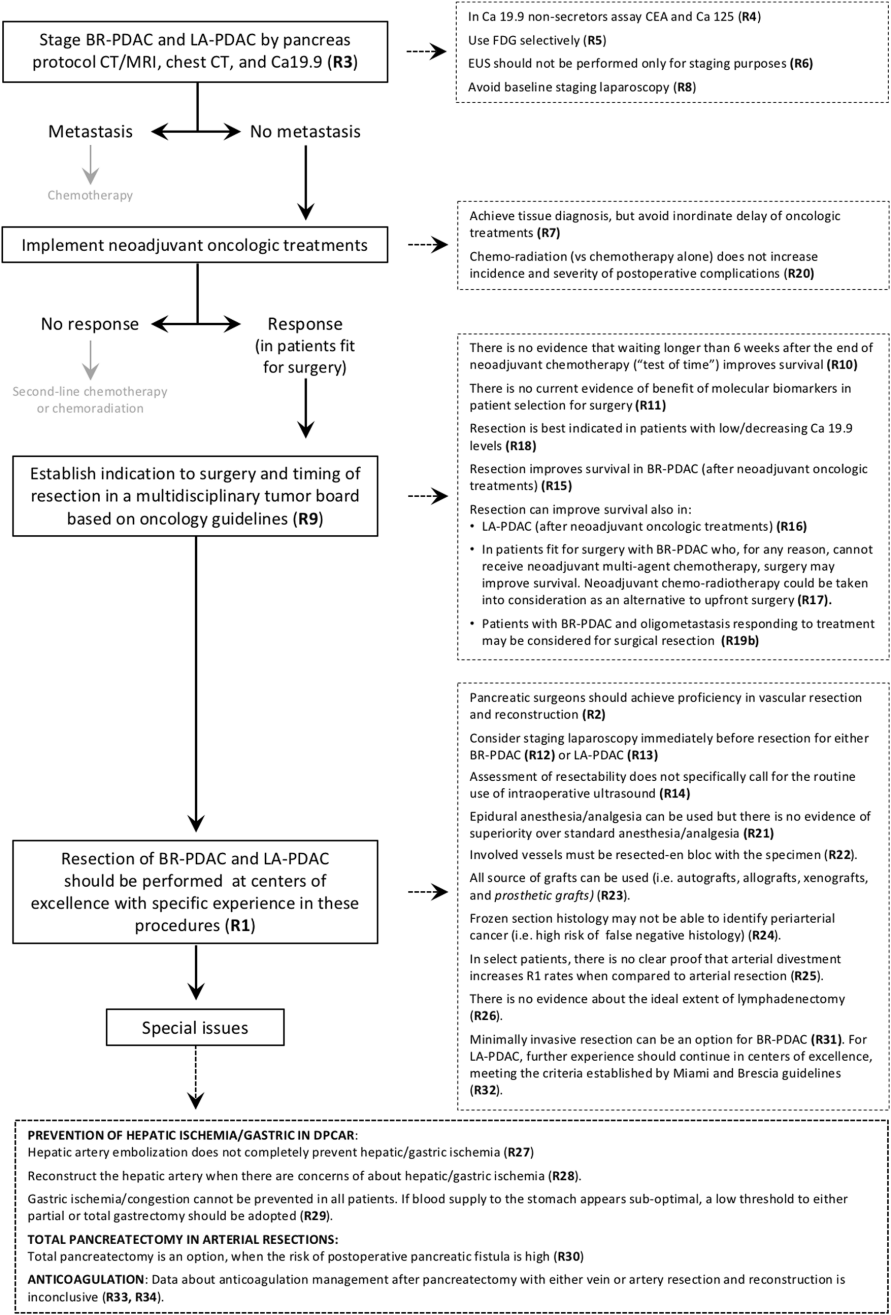
Management algorithm for patients with BR-PDAC and LA-PDAC based on the REDISCOVER guidelines

Although all recommendations received consensus after the online Delphi rounds, only 34 were ultimately endorsed. Twelve distinct clinical questions were amalgamated into 6, while 12 recommendations were dismissed. Among these, three were discarded by the assembly, and nine were rejected by the validation committee. Table 1 delineates the 34 approved recommendations. Notably, 85% of the clinical questions (29 out of 34) were supported by low-level evidence. Consequently, the strength of the recommendations predominantly relied on expert opinion (22 times), followed by weak (10 times), and strong (2 times, one of which was upgraded by experts) evidence. Figure 3 illustrates the management algorithm derived from the 34 validated recommendations.

**Table 1:** REDISCOVER recommendations (from Ann Surg. 2024 Feb 26. 10.1097/SLA.0000000000006248)

		Recommendations	LoE	SoR	Expert agreement%	Audience agreement%	Quality score%
1	Centralization	There are no specific criteria to identify institutions for the centralization of BR and LA-PDAC, however, there is good evidence to support volume–outcome interaction in pancreatic surgery. Patients requiring pancreatectomy with vascular resection should be centralized to centers of excellence with specific experience in these procedures. Patients should be enrolled in prospective database and/or registries	Low	Expert opinion	96	93	73
2	Vascular resection	Vascular resection and reconstruction is a component of contemporary pancreatic surgery. Pancreatic surgeons should achieve proficiency in vascular resection and reconstruction	Low	Expert opinion	95	97	73
3	Staging of BR- and LA-PDAC	The clinical staging of patients with BR and LA PDAC should include pancreas protocol CT/MRI in addition to CT of the chest and baseline Ca19.9	Low	Expert opinion	92	94	69
4	Ca 19.9 non-secretors	In these patients baseline CEA and Ca 125 may be useful	Moderate	Weak	93	94	67
5	FDG-PET for BR- and LA-PDAC	There is no specific role for routine FDG-PET in BR- and LA-PDAC. However, FDG-PET can be selectively employed in patients at higher risk of occult metastasis and to permit evaluation of metabolic response following preoperative oncology treatments	Low	Weak	97	97	76
6	Endoscopic ultrasonography without biopsy	While most patients with BR- and LA-PDAC undergo preoperative EUS to achieve tissue cytology/histology, there is no evidence that EUS should be performed only for staging purposes	Low	Expert opinion	91	99	76
7	Pretreatment biopsy	Tissue diagnosis should be obtained in patients with BR- and LA-PDAC before preoperative oncology treatments. Inordinate delay of treatment should be avoided	Low	Expert opinion	96	99	74
8	Baseline staging laparoscopy	Baseline staging laparoscopy is not advised as a routine. Staging laparoscopy can detect occult metastases in selected patients	Low	Expert opinion	96	97	73
9	Timing of surgery after neoadjuvant treatments	There is no evidence about the optimal timing for surgical resection in patients with BR- or LA-PDAC following neoadjuvant chemo ± radiation therapy. Following NCCN guidelines which indicate that surgery should be performed 4 to 8 weeks after completion of chemotherapy is recommended. All patients should have their case discussed at multidisciplinary tumor board	Low	Expert opinion	98	97	74
10	Delaying surgery (“test of time”)	There is insufficient evidence to recommend waiting longer than 6 weeks after the end of neoadjuvant chemotherapy. Multidisciplinary tumor board discussion should recommend the best timing for surgical resection in individual patients	Low	Expert opinion	99	94	62
11	Molecular biomarkers in patient selection for surgery	There is currently no evidence of benefit from molecular biomarkers in patient selection for surgery. However genetic testing for inherited mutations and molecular tumor profiling is advised	Low	Weak	99	98	69
12	Staging laparoscopy after neoadjuvant treatments (BR-PDAC)	In patients with BR-PDAC, staging laparoscopy may be recommended prior to pancreatic resection if there is suspicion of occult metastases or unresectability	Low	Weak	91	96	76
13	Staging laparoscopy after neoadjuvant treatments (LA-PDAC)	In patients with LA-PDAC, staging laparoscopy is advised prior to laparotomy	Low	Weak	91	90	75
14	Intraoperative ultrasounds	The assessment of resectability of BR-PDAC and LA-PDAC resectability following neoadjuvant therapies does not specifically call for the routine use of intraoperative ultrasound. Intraoperative ultrasound can be used to define anatomy	Low	Expert opinion	97	94	69
15	Pancreatic resection after neoadjuvant treatment (BR-PDAC)	In patients fit for surgery with BR-PDAC, surgical resection improves survival	Moderate	Strong*	93	87	72
16	Pancreatic resection after neoadjuvant treatment (LA-PDAC)	In the absence of progression with good biological response complete surgical resection should be considered to improve survival. All patients should be discussed at a multidisciplinary tumor board. Only centers of excellence should perform these surgeries	Low	Weak	84	82	74
17	Pancreatic resection without neoadjuvant treatment (BR-PDAC)	In patients fit for surgery with BR-PDAC who, for any reason, cannot receive neoadjuvant multi-agent chemotherapy, surgery may improve survival. Neoadjuvant chemoradiotherapy could be taken into consideration as an alternative to upfront surgery. All patients should be discussed at multidisciplinary tumor board. Only centers of excellence should perform these surgeries	Low	Weak	88	81	67
18	Pancreatic resection after neoadjuvant treatments and rising Ca 19.9 levels (BR-PDAC)	Rising Ca19.9 is considered a significant adverse prognostic factor for early recurrence after resection. All patients should be discussed at multidisciplinary tumor board. Only centers of excellence should perform these surgeries	Low	Expert opinion	97	89	76
19	Pancreatic resection after neoadjuvant treatments and oligometastic disease (BR-PDAC)	(a) Oligometastatic disease that develops during neoadjuvant therapy should be considered progression of disease and surgery should not be performed (b) Patients with synchronous oligometastatic disease who receive neoadjuvant therapy and show a good response may be considered for surgical resection in very selected cases and after discussing with patient and family. All patients should be discussed at multidisciplinary tumor board. Only centers of excellence should perform these surgeries	Low	Expert opinion	91	92	69
20	Neoadjuvant chemo-radiation and postoperative complications	There is no evidence that chemo-radiation increases incidence and severity of postoperative complications compared to chemotherapy alone in patients with BR-PDAC undergoing pancreatic resection	Low	Weak	96	95	80
21	Epidural anesthesia/analgesia	Epidural anesthesia can be used. There is no evidence of superiority over standard anesthesia/analgesia	High	Strong	96	93	69
22	En bloc resection of tumor and involved vessels	Attempting en-bloc resection is an established oncologic principle and should be followed	Low	Expert opinion	92	91	63
23	Grafts/patches for vascular reconstruction	Autologous grafts (either vessels or peritoneum), allografts (usually vessels), xenografts (usually bovine pericardium), and prosthetic grafts can all be used for vascular reconstruction at the time of pancreatectomy depending on availability, type of reconstruction, and surgeon preference	Moderate	Weak	97	99	68
24	Frozen section of periarterial tissues	There is insufficient evidence to define the value of frozen section histology of periarterial tissues when discriminating between cancer invasion and perivascular fibrosis. Positive frozen section histology can be employed to decide to proceed with vascular resection or to abort the procedure	Low	Weak	98	96	71
25	Arterial divestment	In BR-PDAC and LA-PDAC, there is no clear proof that arterial divestment increases R1 rates when compared to arterial resection	Low	Expert opinion	96	93	62
26	Lymphadenectomy	There is no evidence to support what an optimal lymphadenectomy is in BR-PDAC and LA-PDAC	Low	Expert opinion	95	97	64
27	Hepatic artery embolization in DPCAR	Embolization of the common hepatic artery, in preparation for distal pancreatectomy with en-bloc resection of the celiac trunk, does not completely prevent hepatic and/or gastric ischemia	High	Strong	93	90	69
28	Hepatic artery reconstruction in DPCAR	The hepatic artery should be reconstructed when there are concerns of developing hepatic ischemia. However there is minimal evidence to define when the common hepatic artery should be reconstructed in a distal pancreatectomy with en-bloc resection of the celiac trunk	Low	Expert opinion	93	90	69
29	Gastric ischemia in DPCAR	In patients requiring total pancreatectomy with en-bloc resection of the celiac trunk gastric ischemia cannot be prevented in all patients. Surgeons should also be aware of venous congestion. If blood supply to the stomach appears sub-optimal, a low threshold to either partial or total gastrectomy should be adopted	Low	Expert opinion	96	95	60
30	Total pancreatectomy and artery resection	Total pancreatectomy is an option in selected patients, particularly when the risk of of pancreatic fistula is felt to be high. Surgeons performing arterial resection should register outcomes into prospective database and/or registries	Low	Expert opinion	95	90	69
31	Minimally invasive surgery in BR-PDAC	There is a role for minimally invasive pancreas resection in BR-PDAC. Further experience should continue in centers of excellence, meeting the criteria established by Miami and Brescia guidelines. Patients should be enrolled in prospective database and/or registries	Low	Expert opinion	96	83	62
32	Minimally invasive surgery in LA-PDAC	There is a very limited role for minimally invasive pancreas resection in LA-PDAC. Further experience should continue in centers of excellence, meeting the criteria established by Miami and Brescia guidelines. Patients should be enrolled in prospective database and/or registries	Low	Expert opinion	92	80	58
33	Anticoagulation in vein resection	Data about anticoagulation management after pancreatectomy with vein resection and reconstruction are inconclusive	Low	Expert opinion	99	100	64
34	Anticoagulation in artery resection	Data about anticoagulation management after pancreatectomy with artery resection and reconstruction are sparse and inconclusive	Low	Expert opinion	97	100	65

*LoE* Level of evidence, *SoR* strength of recommendation, *DPCAR* distal pancreatectomy with resection of the celiac artery

*Upgraded by experts

Table 2 enumerates the 12 recommendations that failed to gain approval. These recommendations addressed eight pivotal areas:

1. Resection and reconstruction of the superior mesenteric artery.
2. Management strategies for LA-PDAC patients fit for surgery unable to undergo multi-agent neoadjuvant chemotherapy.
3. Management of LA-PDAC patients fit for surgery exhibiting elevated serum Ca 19.9 levels post-neoadjuvant oncology treatments.
4. Optimal number of chemotherapy cycles pre-surgery.
5. Comparative efficacy of primary chemoradiotherapy versus chemotherapy alone in LA-PDAC.
6. Appropriate timing for surgical resection post-neoadjuvant/primary chemoradiotherapy
7. Role of ablation therapies.
8. Management of patients with oligometastasis and LA-PDAC.

**Table 2:** Clinical questions discarded from the REDISCOVER guidelines (from Ann Surg. 2024 Feb 26. 10.1097/SLA.0000000000006248)

	LoE	SoR	Expert agreement%	Audience agreement%
Discarded after audience discussions and voting				
Following neoadjuvant treatments, in patients with radiologic encasement of the superior mesenteric artery does tumor resection improve survival when compared to continued medical treatments?				
In the absence of progression with good biological response complete surgical resection should be considered to improve survival. All patients should be discussed at MDT board. Only high-volume centers should perform these surgeries	Low	Expert opinion	88.1	72
In patients fit for surgery with non-metastatic LA-PDAC involving the superior mesenteric artery who, for any reason, cannot receive preoperative multi-agent chemotherapy, does surgery improve survival when compared to alternative treatments?				
In patients with non-metastatic LA-PDAC involving the superior mesenteric artery who are fit for surgery but, for any reason, are unable to receive preoperative multi-agent chemotherapy, chemoradiotherapy should be considered as an alternative to upfront surgery. Given the high level of complexity involved in these procedures, upfront surgery should generally be avoided in these patients. If R2 resection may be avoided, pancreatectomy with resection and reconstruction of the superior mesenteric artery may be carefully evaluated in centers with specific experience and positive postoperative outcomes	Low	Weak	80.2	55
In patients with LA PDAC who received neoadjuvant medical treatments and are fit for surgery but have oligometastic disease, do continued medical treatments improve survival when compared to tumor resection?				
In patients with LA-PDAC who received neoadjuvant medical treatments and are fit for surgery but have oligometastic disease, there is no evidence that resection improves survival when compared continued medical treatments. The best approach to oligometastasis in PDAC is determined by a variety of factors, including oncology and patient characteristics. In some patients with oligometastasis who responded to multi-agent chemotherapy, preliminary data suggest that tumor resection may be beneficial, particularly when tumor markers showed a clear decline, patients were in good clinical condition, and resection of the primary tumor aimed to local radicality. The option of resection should be carefully discussed in a multidisciplinary tumor board considering also the burden of surgery, candidly presented to patients, and documented in a written informed consent. Patients should be closely monitored, and outcome information should be entered into prospective databases	Low	Weak	85.1	67
Discarded by the validation committee				
In patients fit for surgery with non-metastatic LA-PDAC involving the celiac trunk who, for any reason, cannot receive preoperative multi-agent chemotherapy, does surgery improve survival when compared to alternative treatments?				
Chemo-radiotherapy should be taken into consideration instead of upfront surgery in patients with non-metastatic LA-PDAC involving the celiac trunk who are fit for surgery but, for any reason, are unable to receive preoperative multi-agent chemotherapy. In high-volume centers, upfront surgery may be carefully considered if R2 resection can be avoided	Low	Weak	86.1	81
What is the best timing for surgical resection in patients with BR- or LA-PDAC who received primary/neoadjuvant chemo-radiation?				
There is no clear evidence about the best timing of surgery in patients with BR- or LA-PDAC following primary/neoadjuvant chemo-radiation. However, delaying surgery > 10 or > 20 weeks, while adding a short course of additional chemotherapy, can improve pathologic response	Low	Weak	81.2	80
Is there an ideal number of chemotherapy cycles before surgery?				
There is no clear evidence about the ideal number of chemotherapy cycles before surgery. While more preoperative chemotherapy cycles could prolong survival, the decision when chemotherapy is completed and the patient can be considered for surgery, should be taken on an individual basis by a multidisciplinary pancreas tumor board	Low	Weak	97	95
In patients with BR-PDAC undergoing pancreatic resection, does neoadjuvant chemo-radiation improve oncologic outcomes compared to chemotherapy alone?				
Chemo-radiation does not appear to improve oncologic outcomes of patients with BR-PDAC undergoing pancreatic resection, despite higher rates of R0 resection and improved pathological response	High	Strong	91.1	92
In patients with LA-PDAC, does primary chemo-radiation improve oncologic outcomes when compared to chemotherapy alone?				
Currently available data do not fully support the hypothesis that chemo-radiation improves oncologic outcomes of LA-PDAC when compared to chemotherapy alone. Well-designed randomized control trials are required to answer this question	Low	Weak	92.1	91
In patients with BR-PDAC who are fit for surgery, do ablation therapies improve oncologic outcomes compared to pancreatic resection?				
No study has compared ablation therapies to surgery in patients with BR-PDAC fit for surgery. Therefore, at the present time, there is no evidence supporting the hypothesis that ablation therapies could improve oncologic outcomes compared to pancreatic resection	Low	Weak	92.1	98
In patients with LA-PDAC who are fit for surgery, do ablation therapies improve oncologic outcomes compared to pancreatic resection?				
Currently available studies have a retrospective design and are at high risk of selection bias. Therefore, there is no evidence that ablation therapies can improve oncologic outcomes compared to pancreatic resection in patients with LA-PDAC. Preliminary data suggest that ablation therapies could be worth of further investigation	Low	Weak	91.1	96
In patients with LA PDAC who received primary/neoadjuvant medical treatments, are fit for surgery, and have no evidence of distant metastasis but show rising Ca 19.9 levels do continued medical treatments improve survival when compared to tumor resection?				
There is no evidence that continued medical treatments improve survival when compared to tumor resection in patients with LA-PDAC who received neoadjuvant medical treatments, are fit for surgery, and have no evidence of distant metastasis but show rising Ca 19.9 levels. Response of Ca 19.9 to neoadjuvant medical treatments provides relevant prognostic information and is used to select surgical candidates. Probably because of this background, the literature does not provide specific information. Whether or not these patients could be offered resection (after chemotherapy switch), should be carefully defined in a multidisciplinary pancreatic tumor board. Potential advantages of pancreatic resection should be carefully balanced against predictably high postoperative morbidity and mortality rates	Low	Weak	94.1	89
In patients requiring resection and reconstruction of the celiac trunk/hepatic artery and the superior mesenteric artery, that typically includes also resection and reconstruction of the superior mesenteric-portal vein, does total pancreatectomy improves postoperative outcomes when compared to partial pancreatectomy?				
Partial pancreatectomy is barely ever feasible in patients undergoing pancreatectomy with simultaneous resection and reconstruction of the celiac trunk/hepatic artery and the superior mesenteric artery. In this specific setting, total pancreatectomy facilitates both venous and arterial reconstruction	Low	Weak	96	88

*LoE* Level of evidence, *SoR* strength of recommendation

Furthermore, insights from literature reviews and deliberations during the consensus meeting highlighted the need to redefine the current anatomic-based definition of LA-PDAC. This revision aims to align with the emerging concept of prognosis-based resectability and conform to the A, B, C paradigm of borderline resectability.

Post-consensus, the REDISCOVER registry was initiated to amass comprehensive global data. Accessible at https://rediscover.unipi.it/, this registry acts as a pivotal platform for ongoing research and developmental initiatives in this domain. Its primary ambition is to collate an exhaustive dataset focusing on BR-PDAC and LA-PDAC. The REDISCOVER registry invites researchers and healthcare practitioners to contribute vital data, fostering collaborative endeavors to enhance comprehension, treatment modalities, and outcomes for patients afflicted with BR-PDAC and LA-PDAC.

## Discussion

Recently, the REDISCOVER guidelines were released [14]. They provide the first recommendations for the perioperative care of patients with BR-PDAC and LA-PDAC. In this report, the REDISCOVER recommendations were arranged to create a management algorithm based on the progression of clinical decisions. During the REDISCOVER consensus conference, some disruptive concepts were addressed and approved; however, several were either rejected or deemed to be at a nascent stage and early to be included in the guidelines. These important topics are covered in this article.

In general, the REDISCOVER guidelines are based on a low level of evidence thus highlighting the urgent need for further high-quality research. At least in part, the low level of evidence is explained by many studies reporting on BR-PDAC and LA-PDAC as a unique entity. Discussions at the consensus conferences also demonstrated that current definitions of BR-PDAC and LA-PDAC are rather subjective and lack clear prognostic implications. Possibly, newer definitions of BR-PDAC and LA-PDAC should be provided that best match the dynamic view of PDAC "stage" based on response to primary and neoadjuvant oncology treatments.

Indeed, the primary message from the REDISCOVER guidelines is that the more dynamic and, to some extent, logical concept of tumor biology predicting prognosis has superseded the static paradigm of vascular involvement as a marker of poor prognosis/unresectability. On the other hand, our understanding of PDAC biology is still incomplete. Chemotherapy response is currently employed as a surrogate marker of good tumor biology; nevertheless, some patients who appear to respond well to oncology therapies still have early tumor recurrence and are unlikely to benefit from radical resection. The use of molecular biomarkers appears to be the most sensible development of the biological selection theory [15]. However, because of the current low probability of obtaining key prognostic information and the high costs, routine molecular testing cannot be advised in current clinical practice. While the NCCN guidelines currently recommend molecular profiling in LA-PDAC, the probability to identify potentially actionable somatic mutations is quite low and most public health systems do not cover the costs of molecular testing. On practical grounds, only BRCA testing has a real chance to impact oncology decisions, but has no clear implications in the selection of surgical candidates. BRCA mutations are identified in 5%-7% of Caucasian patients [16]. These patients are more susceptible to treatment with platinum compounds and poly (ADP-ribose) polymerase inhibitors [17]. In addition, a study in patients with metastatic PDAC and germline BRCA mutations showed that maintenance treatment with olaparib versus placebo improved median progression-free survival in patients who had stable disease after a 16-week platinum-containing chemotherapy regimen. However, median overall survival was not affected [18]. Therefore, outside clinical trials, molecular testing should be reserved for high-risk individuals and patients with a family history of PDAC for the purpose of genetic counseling [19]. Identification of reliable prognostic markers for the selection of surgical candidates is a main target of future research projects.

In light of the REDISCOVER guidelines, the need to resect peripancreatic vessels after neoadjuvant oncology treatments should be mainly considered a marker of technical difficulty without clear prognostic implications. While adding further technical complexity to pancreatectomy increases the incidence and severity of postoperative complications, several recent studies have shown improved results even in patients requiring arterial resection [20–24]. It is important to underscore here that ensuring acceptable postoperative results in the context of BR-PDAC and LA-PDAC does not only require the ability to perform vascular reconstructions. It rather entails additional technical skills that begin with preoperative planning and end up with a wide range of intraoperative strategies aiming to provide a safe approach to target vessels while respecting the golden principles of surgical oncology and minimizing surgical trauma in terms of intestinal and hepatic ischemia, bowel congestion, and intraoperative bleeding. While vascular reconstruction can be left to either vascular or transplant surgeons, the other tasks require specific skills. Therefore, patients with BR-PDAC and LA-PDAC should be centralized to centers with specific experience in these procedures. The REDISCOVER guidelines defined these institutions as centers of excellence. A recent Scandinavian study demonstrated that a center with a recruitment area of approximately 3 million is expected to manage approximately 75–80 patients with BR-PDAC and LA-PDAC per year, leading to approximately 15 resections for BR-PDAC and 5 for LA-PDAC per year [13]. Pancreatic surgery is sensitive to the effects of centralization. Figures from the Scandinavian study further reinforce the importance of centralization for BR-PDAC and LA-PDAC.

It is crucial to underscore that there comes a point where technical complexity, the patient's physiological status, and the prognostic outlook must be considered collectively. In other words, when anticipating high surgical difficulty in patients with less than optimal performance status and/or tumors exhibiting intermediate biology, the decision to proceed with tumor resection should be discouraged regardless of technical feasibility. These factors should be thoroughly discussed and openly weighed when obtaining informed consent for resection.

### Resection and reconstruction of the superior mesenteric artery

The REDISCOVER guidelines recommend LA-PDAC resection in carefully selected patients. However, pancreatectomy with resection and reconstruction of the superior mesenteric artery (PRR-SMA) could not be recommended because of lack of consensus after the audience vote (72%). The majority of experts had agreed on PRR-SMA on the online Delphi rounds (88%).

There is no evidence that involvement of the superior mesenteric artery portends a worse prognosis when compared to the same degree of local tumor spread around the celiac trunk [25]. In fact, following neoadjuvant treatments, there is even no evidence that the prognosis of LA-PDAC is inferior to that of BR-PDAC, further reinforcing the concept of prognosis-based resectability [13, 25]. Most audience's concerns regarding PRR-SMA regarded the high level of technical difficulty of this procedure that typically leads to high rates of morbidity and mortality.

PRR-SMA is clearly a complex procedure, but postoperative outcomes are rapidly improving because of technical refinements and growing experience [20]. Two recent studies from China proposed the adoption of intestinal autotransplantation to overcome the challenges of PRR-SMA. Combining the data of these two studies, 46 PRR-SMAs were performed. Severe postoperative complications occurred in 17 patients (37%) and two patients died (4.3%) [23, 24]. In a recent Western study, 95 PRR-SMA were reported from a single center. In 91 and 32 patients, respectively, the superior mesenteric vein (96%) and the celiac trunk/hepatic artery (34%) were also resected. Upon completion of the learning curve (37 procedures) 3 of 58 patients died within 90 days (5.2%) [21]. These data favorably compare with the prohibitive mortality (11.8%) reported, only 10 years ago, for all types of pancreatectomy with arterial resection (11.8%), as well as, with a more recent systematic review on 70 PRR-SMA (20%) [26, 27].

Further experience with PRR-SMA should continue, beyond the REDISCOVER guidelines, only in centers with specific skills and experience. Data should be recorded in international registries, such as the REDISCOVER registry, or reported in prospective observational studies. Diffusion of PRR-SMA is unlikely to occur quickly, but denying resection solely because of lack of surgical experience should be carefully considered. While proficiency is progressively gained in relatively less complex procedures, consideration should be given to refer these patients to expert centers.

### Management strategies for LA-PDAC patients fit for surgery unable to undergo multi-agent neoadjuvant chemotherapy

Most data concerning the survival advantage of neoadjuvant chemotherapy refer to multi-agent chemotherapy [28]. In a recent Scandinavian study approximately 20% of the patients with BR-PDAC and LA-PDAC could only receive best supportive care. FOLFIRINOX was delivered to only 50% of the patients while 15% received single-agent chemotherapy with gemcitabine. In the FOLFIRINOX group 53% of the patients suffered grade 3–5 adverse events and two of them died (1.9%) [13]. In an intention-to-treat study, 216 of 254 patients (85.0%) experienced FOLFIRINOX-related toxicity. Grade 3–4 toxicity was documented in 109 patients (42.9%), 100 patients required inpatient admission and management, while 73 patients (28.7%) required an emergency department admission. Poor tolerability (46.3%) was the main reason for not completing the 8 planned cycles of FOLFIRINOX [12]. The probability to receive multi-agent chemotherapy is mostly influenced by age and performance status. Only 10% of the patients aged 75 years or older can receive FOLFIRINOX and less than half of them complete the treatment [29]. More than half of patients receiving FOLFIRINOX require a biliary stent and almost a third of them requires additional endoscopic interventions for obstructed stents and/or cholangitis resulting in treatment delay and/or dose reduction. Overall, over 20% of the patients who are initiated on FOLFIRINOX fail to complete the number of planned cycles [12]. The possibility to receive FOLFIRINOX chemotherapy is not influenced by local tumor status [12, 29].

Therefore, it is clear that not all patients can receive multi-agent primary/neoadjuvant chemotherapy. Some of these patients, however, may be fit for surgery. The REDISCOVER guidelines acknowledged that upfront surgery may improve survival in BR-PDAC when multi-agent neoadjuvant chemotherapy cannot be delivered, but could not provide a similar recommendation for LA-PDAC. In these patients, efforts should be maximized to permit delivery of multi-agent neoadjuvant chemotherapy, failing which proceeding with resection does not appear to provide a clear oncological advantage.

### Management of LA-PDAC patients fit for surgery exhibiting elevated serum Ca 19.9 levels post-neoadjuvant oncology treatments

The probability of radical resection is predicted by the Ca 19.9 level, which carries clear prognostic implications in PDAC [30, 31]. Ca 19.9 levels of > 500 kU/L are a biologic factor associated with borderline resectability [9]. Long-term survival following resection is predicted by both a decrease in Ca 19.9 of ≥ 50% and a normalization of Ca 19.9 in response to neoadjuvant oncology therapies [32, 33]. Depending on pretreatment levels, between 66 and 22% of patients achieve normalization of Ca 19.9 levels [32]. The best indicators to anticipate favorable survival are a baseline level of Ca 19.9 < 80 kU/L and a response to treatment of ≥ 85% [33, 34]. Predicting post-resection outcomes is further improved by Ca 19.9 dynamics during oncology treatments [35].

However, following neoadjuvant oncology therapies, Ca 19.9 level does not decrease or rises in approximately 10% of patients [33]. Some of these patients are fit for surgery, have no evidence of distant metastasis, and harbor a potentially resectable tumor. If “high” Ca 19.9 levels persist following chemotherapy switch, the surgeon is faced with the difficult dilemma of denying resection based only on Ca 19.9 levels. In these patients, according to oncology guidelines, the most sensible course of action is radiation treatment [15, 19]. However, the REDISCOVER guidelines recommended resection for BR-PDAC with stable/rising Ca 19.9 levels but denied this possibility for LA-PDAC. Considering LA-PDAC and BR-PDAC share the same biology, once again, technical complexity and higher operative risk were the main reasons to deny resection in LA-PDAC in the absence of favorable Ca 19.9 response.

### Optimal number of chemotherapy cycles pre-surgery

There is no agreement about the ideal number of cycles of chemotherapy before resection. The 2024 NCCN guidelines recommend ≥ 2 to 6 cycles of gemcitabine plus cisplatin in BRAC mutated patients, and ≥ 4 to 6 cycles of all the other chemotherapy regimens (namely, FOLFIRINOX, m FOLFIRINOX, NALIRIFOX, and gemcitabine plus albumin-bound paclitaxel) [15]. The 2023 ESMO guidelines do not recommend a specific number of cycles [19]. A recent, phase 2, randomized and controlled trial employed 8 cycles of mFOLFIRINOX as a neoadjuvant chemotherapy regimen for BR-PDAC and found that this regimen was superior to 7 treatment cycles of mFOLFIRINOX followed by stereotactic body radiotherapy or hypofractionated image-guided radiotherapy [36]. The ESPAC5 trial compared different short-course neoadjuvant oncology regimens (gemcitabine plus capecitabine: two cycles; FOLFIRINOX: four cycles; and capecitabine-based chemoradiotherapy: capecitabine 830 mg/m2 twice a day orally over the 5.5 weeks of radiotherapy) versus upfront surgery in BR-PDAC. Neoadjuvant chemotherapy (either gemcitabine plus capecitabine or FOLFIRINOX) had the best survival compared with upfront surgery [37].

An international cohort study of 520 patients evaluated adjuvant chemotherapy in patients with resected pancreatic cancer after at least 2 cycles of neoadjuvant FOLFIRINOX treatment (47% resectable PDAC; 40% BR-PDAC; 10% LA-PDAC; 3% stage unknown). The median number of neoadjuvant FOLFIRINOX cycles was 6 for patients who received adjuvant therapy and for those who did not [38]. In a recent systematic review and meta-analysis, the median number of FOLFIRINOX cycles administered to with LA-PDAC ranged from 4.9 to 11.5. The number of FOLFIRINOX cycles did not influence the rate of surgical resection and R0 resection [39]. In a similar study on BR-PDAC the median number of FOLFIRINOX cycles was ranged from 4 to 9. The median number of chemotherapy cycles did not affect overall survival [40].

In a systematic review and meta-analysis on neoadjuvant gemcitabine plus nab-paclitaxel in BR-PDAC and LA-PDAC, the median number of chemotherapy cycles ranged from 2 to 8 [41]. In a single-center retrospective study the median number of neoadjuvant gemcitabine plus nab-paclitaxel for BR-PDAC was 3 (range 1–10) [42]. In the recent NORPACT-2 trial, the number of neoadjuvant chemotherapy cycles was 4 for FOLFIRINOX and 2 for gemcitabine plus nab-paclitaxel [13].

It is evident that we lack clarity regarding the ideal number of chemotherapy cycles to administer before surgery in patients with BR-PDAC and LA-PDAC. Moreover, the influence of dose reductions and the extent of dose reduction on the identification of suitable surgical candidates remains uncertain. There is an urgent need for further research to address these critical questions.

### Comparative efficacy of primary chemoradiotherapy versus chemotherapy alone in LA-PDAC

Primary chemotherapy is typically favored in LA-PDAC due to its ability to achieve systemic disease control and potentially induce downstaging of the primary tumor. However, it may not adequately address local disease control in all patients.

Primary chemoradiotherapy combines the cytotoxic effects of chemotherapy with the locoregional control provided by radiotherapy. In theory, it should be beneficial for LA-PDAC where achieving local disease control is a priority. Studies evaluating primary chemoradiotherapy in LA-PDAC have shown promising results in terms of local tumor response, downstaging, and achieving negative surgical margins. However, chemoradiotherapy does not seem to improve overall survival. A meta-analysis of 5 randomized controlled trials revealed that chemoradiotherapy did not confer a survival advantage compared to chemotherapy alone but increased the rates of grade 3 to 4 adverse events [43, 44].

Some patients receive primary chemotherapy followed by consolidation radiotherapy. Although these patients are not initially considered surgical candidates, some of them may eventually undergo surgery due to stable disease and good clinical conditions. However, surgery in these patients is technically more complex due to the consolidation of radiotherapy effects into retroperitoneal scarring tissue. Whenever possible, radiotherapy should be used for neoadjuvant purposes.

The choice of oncological treatment in LA-PDAC is often based on the practices and preferences of individual institutions. Therefore, defining the optimal treatment pathway is a key research objective.

### Appropriate timing for surgical resection post-neoadjuvant/primary chemoradiotherapy

The optimal timing for surgical resection in patients with BR-PDAC or LA-PDAC following neoadjuvant or primary chemoradiotherapy is still a topic of debate and ongoing research.

A study demonstrated that prolonging the interval between completion of chemoradiotherapy and surgery, with continued chemotherapy, for up to 20 weeks was linked with several benefits. These included an enhanced pathologic response and an extended median overall survival [45].

However, despite these findings, consensus has yet to be reached regarding the precise timeframe for surgery after chemoradiotherapy in these patient populations. Consequently, determining the optimal timing for surgical intervention after chemoradiotherapy remains a significant research objective.

### Role of ablation therapies

Different ablation techniques have been developed and proposed especially for LA-PDAC. Non-thermal ablation techniques include irreversible electroporation, stereotactic body radiation, photodynamic therapy, and brachytherapy. Thermal ablation therapies include high-intensity focused ultra-sound, cryoablation, radiofrequency ablation, microwave ablation, and laser-induced thermotherapy. Irreversible electroporation holds significant promise; however, to date, all of these approaches are still considered investigational and lack an established role in the management of BR-PDAC and LA-PDAC [46, 47].

### Management of patients with oligometastasis and LA-PDAC

PDAC often exhibits early metastatic dissemination. Initially, metastases may be microscopic and undetectable. Once metastases become visible, regardless of their number, the disease is considered systemic, and treatment typically revolves around chemotherapy.

The concept of oligometastasis has emerged as a result of recent advancements in oncology. In this scenario, the number and sites of metastases are limited making localized cancer treatments of potential benefit. Examples include liver metastases from colorectal cancer, lung metastases from various primary tumors, and adrenal metastases from lung cancer [48]. However, a clear definition specifying the maximum number of metastases qualifying as oligometastasis is lacking.

In PDAC, metastases are primarily found in the liver, peritoneum, or lungs. Lung metastases may exhibit a less aggressive biological behavior. Resection of isolated lung metastases in PDAC is already considered a treatment option for carefully selected patients [49]. In the abdomen, the concept of oligometastasis primarily pertains to liver metastases. Generally, in patients who demonstrate a robust response to chemotherapy over 8–9 months and exhibit no signs of tumor progression shortly after discontinuing chemotherapy, resection of liver metastases alongside the primary tumor is being considered for carefully selected individuals [50].

In LA-PDAC, the concept of oligometastasis has not been extensively explored, likely due to concerns regarding the complexity of surgery. Furthermore, when metastases were initially occult, distinguishing between synchronous metastases that responded to treatment and metastases that developed despite oncology treatments can be challenging.

Future studies should endeavor to establish a clear definition of oligometastasis in PDAC and elucidate its prognostic implications and treatment options, particularly for LA-PDAC.

## Conclusions

The REDISCOVER consensus conference marks a milestone by introducing the first surgical guidelines for BR-PDAC and LA-PDAC. This manuscript presents a management algorithm derived from these guidelines and discusses unresolved clinical questions.

The REDISCOVER guidelines unequivocally mark a shift in the indication for resection of BR-PDAC and LA-PDAC, prioritizing tumor biology over anatomical features as the primary indication for resection. These guidelines also underscore the necessity of revising the anatomical definition of LA-PDAC, as shown by the discrepancy in surgical recommendations based on involvement of the celiac trunk versus the superior mesenteric artery. Furthermore, the current definition of LA-PDAC includes the scenario of an unreconstructible superior mesenteric/portal vein, signifying the tumor as unresectable by definition, regardless of considerations about treatment response and tumor biology. Finally, the new definition should aim to establish a clearer distinction between BR-PDAC and LA-PDAC.

Finally, it is important to refine existing management strategies. The establishment of the REDISCOVER registry (https://rediscover.unipi.it/) holds promise as a unified research platform aimed at advancing our understanding and improving the management of BR-PDAC and LA-PDAC.

## Data Availability

This manuscript provides guidelines on the perioperative care of surgical patients with borderline and locally advanced pancreatic cancer. As such, it followed the methodology required for this type of action that does not require individual patient data. Detailed results of systematic literature reviews can be provided, upon reasonable request.
